# Central nervous system remyelination in culture — A tool for multiple sclerosis research

**DOI:** 10.1016/j.expneurol.2011.04.009

**Published:** 2011-07

**Authors:** Hui Zhang, Andrew A. Jarjour, Amanda Boyd, Anna Williams

**Affiliations:** MS Centre, Centre for Regenerative Medicine, University of Edinburgh, Queen's Medical Research Centre, 47, Little France Crescent, Edinburgh EH16 4TJ, Scotland, UK

**Keywords:** Caspr, contactin-associated protein, CNS, central nervous system, DIV, days *in vitro*, EAE, experimental allergic encephalitis, LPC, lysophosphatidylcholine, MR, magnetic resonance, MS, multiple sclerosis, MBP, myelin basic protein, NFH, neurofilament, OPCs, oligodendrocyte precursor cells, Remyelination, Multiple sclerosis, Oligodendrocyte, Myelination, Demyelination

## Abstract

Multiple sclerosis is a demyelinating disease of the central nervous system which only affects humans. This makes it difficult to study at a molecular level, and to develop and test potential therapies that may change the course of the disease. The development of therapies to promote remyelination in multiple sclerosis is a key research aim, to both aid restoration of electrical impulse conduction in nerves and provide neuroprotection, reducing disability in patients.

Testing a remyelination therapy in the many and various *in vivo* models of multiple sclerosis is expensive in terms of time, animals and money. We report the development and characterisation of an *ex vivo* slice culture system using mouse brain and spinal cord, allowing investigation of myelination, demyelination and remyelination, which can be used as an initial reliable screen to select the most promising remyelination strategies. We have automated the quantification of myelin to provide a high content and moderately-high-throughput screen for testing therapies for remyelination both by endogenous and exogenous means and as an invaluable way of studying the biology of remyelination.

## Graphical abstract

**Figure:**
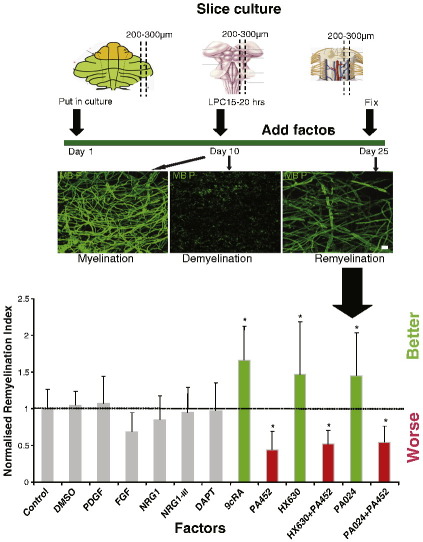

**Highlights:**

► We model central nervous system demyelination and remyelination in a culture system. ► We automate remyelination quantification allowing a moderately high-throughput and high content system. ► We validate this system with factors known to effect remyelination in vivo. ► We have a new tool for studying remyelination for multiple sclerosis research.

## Introduction

Multiple sclerosis (MS) is the commonest cause of disability in young people in the western world after trauma, with Scotland having the highest prevalence of patients in the world (1 in 500 people). The cause of MS is not clear, but its pathology consists of immune infiltration into the central nervous system (CNS), inflammation, demyelination and axonal degeneration (Dubois-Dalcq et al., 2008). The presence of demyelinating plaques in the CNS corresponds to MS relapses, but it is axonal degeneration which correlates with progressive disability (De Stefano et al., 2001; Fisniku et al., 2008). Myelination of axons allows fast saltatory conduction of electrical impulses, and provides support both mechanically and functionally by cellular communication between the axon and the oligodendrocyte which produces the myelin sheath. Demyelination of axons reduces the conduction velocity of nerve impulses, but also makes the axons vulnerable to degeneration (Franklin and Ffrench-Constant, 2008). In MS, some demyelinated plaques contain axons which have been remyelinated, which restores nerve conduction (Duncan et al., 2009; Smith et al., 1981) and protects the nerve from subsequent degeneration (Irvine and Blakemore, 2008). There are now anti-inflammatory immunomodulatory treatments that reduce the relapse rate in MS, but there are no current neuroprotective treatments to reduce progressive disability in MS. Therefore, one neuroprotective strategy is to try to improve the efficiency of remyelination. Oligodendrocyte precursor cells (OPCs) are the effector cells of remyelination and these migrate towards demyelinating MS lesions, proliferate, differentiate and remyelinate axons, but with limited efficiency (Patani et al., 2007; Patrikios et al., 2006; Perier and Gregoire, 1965; Prineas and Connell, 1979; Raine and Wu, 1993), which decreases with the age of the patient and the longevity of the disease (Goldschmidt et al., 2009). Therefore, improvement of the efficiency of remyelination is a major aim within the MS community, and if accomplished is likely to be of great importance clinically. This may be achieved either by enhancing endogenous remyelination or by introducing exogenous cells to stimulate remyelination.

It is not yet possible to follow remyelination reliably in humans, although some progress is being made into obtaining magnetic resonance (MR) sequences which correlate with remyelination, particularly with the use of Magnetisation Transfer Ratio sequences. PET scan ligands are also being developed to identify and quantify the amount of myelin present in areas of the MS brain (Stankoff et al., 2006). In humans, correlation with pathology can only be performed by doing MR scans on post-mortem brains (Barkhof et al., 2003; Schmierer et al., 2009), from rare biopsies (Bruck et al., 1997) or by serendipitous MR scans shortly before a MS patient dies, with subsequent pathology (Chen et al., 2007). Thus, we must use animal models of MS to discover and develop new strategies for enhancing remyelination to reduce axonal degeneration and patient disability.

Currently, most researchers use *in vitro* models of developmental myelination and *in vivo* models of remyelination. *In vitro* systems culturing OPCs with CNS or peripheral nervous system neurones are relatively simple, inexpensive, high-throughput models (Chan et al., 2004; Lubetzki et al., 1993; Wang et al., 2007; Watkins et al., 2008). However, they are models of myelination and not remyelination, which occurs in the presence of inflammation, injury and insult. For this reason, extrapolation of results from *in vitro* models to *in vivo* situations can be unreliable.

*In vivo* models include experimental allergic encephalitis (EAE), focal myelin toxin injection and cuprizone ingestion — reviewed in Blakemore and Franklin (2008) and Furlan et al. (2009). These models each reflect different aspects of the pathology of MS and are the current accepted gold standards for modelling the disease, but these models are very low-throughput, and so expensive in terms of animals, time and money.

A method of culturing *ex vivo* rat organotypic slices for electrophysiological recordings dates back to 1941 (Levi and Meyer, 1941), but myelination was first reported in longer term cerebellar slices in 1956 (Hild, 1956). Demyelination of these slices was achieved as early as 1959, by adding serum from animals with EAE (Bornstein and Appel, 1959). However, the technique was developed further to study myelination when immunohistochemical techniques were fully developed (Notterpek et al., 1993). In 2004, lysophosphatidylcholine (LPC) was used to demyelinate rat cerebellar slices, with the subsequent return of myelin sheaths suggestive of remyelination (Birgbauer et al., 2004). More recently still, our group and others have used this technique to investigate the action of exogenous molecules/drugs on the rate of CNS remyelination (Huang et al., 2011; Mi et al., 2009; Miron et al., 2010).

However, previously, the slice model has never been characterised nor validated. We report the further development of this slice model of CNS remyelination in the mouse cerebellum, brain stem and spinal cord. We fully characterise the model through myelination, demyelination and remyelination, showing that compact myelin is formed, destroyed and replaced, and that remyelinated axons have shorter internodes and thinner myelin. We also have developed an automated system of quantifying (re)myelination, to enable the use of this model as a fast and objective screen. We tested the model with factors known to affect remyelination *in vivo* to determine the fidelity of our automated slice quantification system to the *in vivo* situation and provided proof of principle that exogenous manipulated OPCs added to slices are able to myelinate axons.

## Materials and methods

Animal work was carried out in accordance with the University of Edinburgh regulations under Home Office rules, with local ethical committee consent.

### Slice culture

P1–P2 mouse pups were decapitated, and their brains or spinal cords were dissected into ice-cold Hank's Balanced Salt Solution (HBSS). 200–300 μm sagittal slices of cerebellum, brainstem or spinal cord were cut using a McIlwain tissue chopper. The slices were placed on Millipore Millicell-CM organotypic culture inserts (Fisher) in medium containing 50% MEM with Earle's salts, 25% Earle's Balanced Salt Solution, 25% heat-inactivated horse serum (HIHS), glutamax-II supplement with penicillin–streptomycin, amphotericin B (all purchased from Invitrogen) and 6.5 mg/ml glucose (Sigma). Medium was changed every two days. After 10 days in culture, demyelination was induced by addition of 0.5 mg/ml lysophosphatidylcholine (lysolecithin, LPC, Sigma) to the medium for 15–20 h, after which slices were transferred back into normal medium. Cerebellar slice cultures require around 16 h, whereas brainstem and spinal cord cultures require around 18 h of incubation. Concentrations of LPC higher than this are also toxic to axons. Medium containing factors was added 12 h later. Factors used were Platelet Derived Growth Factor (PDGF) (10 ng/ml, teproTech Inc.), Fibroblast growth factor (FGF) (10 ng/ml, teproTech Inc.), Neuregulin 1 (NRG1) (10 ng/ml, R&D Systems), NRG1-III (10 ng/ml, R&D Systems), DAPT (gamma secretase inhibitor, 5 μM, CalBiochem), 9-cis retinoic acid (9cRA, 50 nM, Sigma), 9cRA agonists HX630 and PA024, and 9cRA antagonist PA452 (1 μM, 500 nM, 5 μM respectively, kindly supplied by Hiroyuki Kagechika). Cultures were maintained for a further 14 days, and then processed for immunolabelling. For proliferation assays, BRDU (Roche) was added for 16 h to the culture medium before fixation at DIV10 (myelination M), DIV12 (demyelination DM) and DIV25 (remyelination RM).

### Immunofluoresence

Slices were fixed while attached to membranes with 4% paraformaldehyde (PFA) in phosphate-buffered saline (PBS) for 1 h, rinsed in PBS for 10 min thrice, and blocked with 3% HIHS, 2% bovine serum albumin (BSA), and 0.5% Triton X-100 in PBS for 1 h. Slices were then incubated in primary antibody overnight, washed once for 10 min and then thrice for 1 h. The slices were then incubated in secondary antibody overnight, washed thrice and mounted. The antibodies Nkx2.2 and PCNA required antigen retrieval before incubation with the primary antibody, by microwaving in Vector H-3300 antigen unmasking solution (Vector Laboratories) for 5 min. Antibodies used include: contactin associated protein (Caspr, mouse, 1 in 500, Abcam), CD68 (mouse, 1 in 200, Serotec), myelin basic protein (MBP, rat, 1 in 500, Serotec), neurofilament (NFH, chick, 1 in 50,000, EnCor), Neuronin N (NeuN, 1 in 300, Chemicon), Nkx2.2 (rabbit, 1 in 200, Developmental Studies Hybridoma Bank, University of Iowa), NG2 (mouse, 1 in 200, Chemicon), Olig2 (goat, 1 in 40, R & D systems), Periaxin (1 in 2000, Repeat Periaxin antibody, kindly supplied by P.J. Brophy), PCNA (rabbit, 1 in 2000, Abcam) and BrdU (mouse, 1 in 500, Chemicon). Appropriate fluorescent secondary antibodies were used (Alexa Fluor, Invitrogen).

### Transmission electron microscopy (EM)

Samples were fixed in 2.5% glutaraldehyde in 0.1 M sodium cacodylate buffer, pH 7.3, for 2 h, washed in PBS and post-fixed in 1% osmium tetroxide in 0.1 M sodium cacodylate for 45 min. The samples were then dehydrated in increasing concentrations of acetone and embedded in Araldite resin. Sections, 1 μm thick, were cut on a Reichert OMU4 ultramicrotome (Leica Microsystems), stained with toluidine blue and viewed in a light microscope to select suitable areas for investigation. Ultrathin sections, 60 nm thick were cut from selected areas, stained in uranyl acetate and lead citrate and then viewed in a Phillips CM120 Transmission electron microscope (FEI). Images were taken on a Gatan Orius CCD camera (Gatan). The perimeter of the axon and the outer border of the myelin sheath was traced and measured with Image Pro Plus software (Media Cybernetics). The G ratio was obtained by dividing these values. The axon diameter was measured by taking the average length of diameters measured at 2° intervals and passing through the centre of the axon. The Maximum Likelihood Test was used to test the likelihood that the two G-ratio regression lines were statistically different.

### Quantification

Confocal microscopy was used to obtain stacks of photographs of MBP and NFH immunolabelling at 1 μm intervals in white matter areas at ×40 magnification. Slices thinned after culture for 25 days to approximately 30 μm thickness. Myelinated fibres are best observed between a depth of 5 μm to 20 μm from the upper surface and all our results were taken from this level. A macro was written with Image Pro Plus software (Media Cybernetics) to automate quantification of the myelination index. The photographs were merged at each level in the stack and the pixels where there was overlap of red and green within a predefined intensity were highlighted forming a mask of co-localization. Very small areas of pixels positive for overlap were excluded automatically to avoid artefact (area under 30 pixels, area of 1 pixel = 72264 nm^2^). Then, the area covered by positive (overlapping) pixels was measured. A similar procedure was performed for NFH staining and the two measurements divided to form the “Myelination Index” — as a measure of the amount of myelin sheath per axon area. The macro written within the programme platform performs all these steps automatically, in around 10 s per image stack. Comparison of the effect of factors on the Myelination Index and proliferation was analysed statistically using ANOVA, followed by the Dunnett's Multiple Comparison Test. Data comparing multiple factors on the Myelination Index is presented normalised to the control group (no additive) which is given the Myelination Index of 1.

Internodal lengths were measured from merges of photographs of slices stained with MBP, NFH and Caspr, and the distance between Caspr staining at paranodes was traced using ImageJ. Comparison of the frequency-distribution of internode length was analysed statistically using the Kolmogorov–Smirnov Test for non-normally distributed data.

### Addition of virally-transduced OPCs to slices

Mouse OPCs were isolated using the shake-off method described previously (McCarthy and de Vellis, 1980) and plated onto poly-d-lysine coated 6 well plates. Lentivirus containing a GFP sequence with a post-transcriptional enhancer under the Cytomegalovirus (CMV) promoter (engineered from Invitrogen pLenti6) and with a Vesicular Stomatitis Virus (VSV) envelope protein, was added to the OPCs at a concentration of 50 particles per cell for 4 h (kindly provided by Pamela Brown). OPCs were maintained in a proliferative state for 2 days with addition of PDGF (10 ng/μl) and FGF (10 ng/μl) and 2% foetal calf serum in Dulbecco's modified Eagles medium. The cells were then trypsinised using TrypLE express solution (Gibco) for 5 min, spun down at 1000 rpm and resuspended in slice culture medium. These cells were pipetted directly onto slices (approximately 10,000 to each slice) and the cultures maintained for 14 days. GFP-containing oligodendrocytes were visible directly with a fluorescent microscope, and the cultures were co-stained for MBP and NFH as described above.

## Results

### Myelination, demyelination and remyelination occurs in slice cultures

**Fig. 1:**
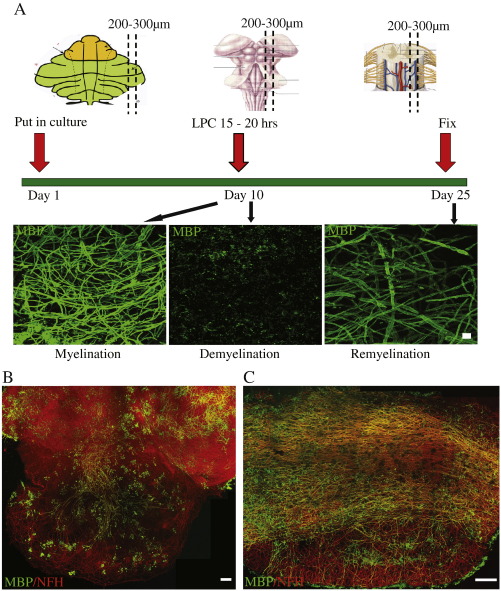
Summary of culture method. A) Slices from cerebellum, brain stem and spinal cord are cultured and allowed to myelinate. By day 10, myelination, as assessed by Myelin Basic Protein (MBP) immunofluorescence (green) is robust. Lysophosphatidyl choline (LPC) is added to the culture medium on day 10, and MBP immunostaining shows only myelin debris. Remyelination is assessed at day 14 after LPC treatment and MBP staining shows the return of myelinated internodes. Scale bar 10 μm. B–C) Cerebellum with attached brainstem (B) and spinal cord (C) showing axons (immunofluorescence to NFH, red) and myelin (immunofluorescence to MBP, green) at 10 DIV. Scale bars 100 μm. (Images taken with ×20 objective and stitched together.)

**Fig. 2:**
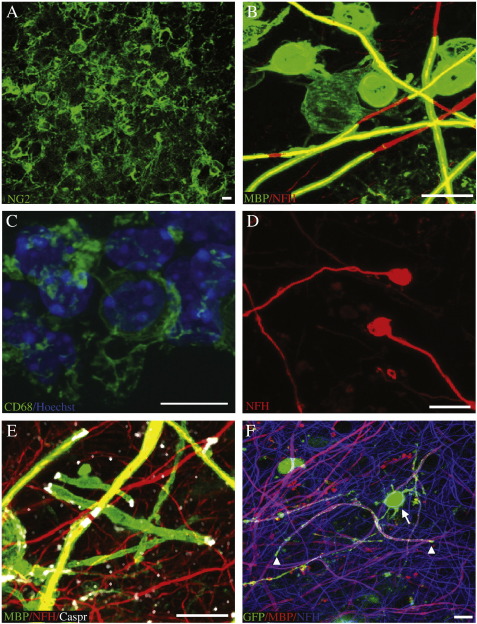
Characterisation of the cells in the model. A) NG2 positive cells (green) show that oligodendrocyte precursor cells (OPCs) are present and remain after demyelination of slices. B) Mature oligodendrocytes (immunofluorescence to MBP, green) remyelinate axons (immunofluorescence to NFH, red) in slice cultures. The photo is overexposed to show the oligodendrocyte arms linking the cell body and myelin sheaths. C) Microglia stained with antibodies to CD68 (green) and Hoechst (blue) showing that resident microglia are present in slices. D) After demyelination, axonal bulbs can be seen, reminiscent of those seen in MS plaques (immunofluorescence to NFH, red.) E) Nodes of Ranvier reform after remyelination (MBP green, NFH red, Caspr, white). F) Exogenous OPCs, transduced with lentiviral vectors to express cytoplasmic GFP, can be added to slices, and are able to myelinate axons. GFP fills the cytoplasmic channels of the myelinating process, whereas MBP antibody stains the myelin sheath GFP green, MBP red, NFH blue). Arrow — GFP + oligodendrocyte cell body. Arrowheads — limit of a myelinated internode seen by the gap in red MBP staining and limit of cytoplasmic GFP staining. Scale bars 10 μm.

Slices of cerebellum, brainstem and spinal cord between 200 and 300 μm in thickness are taken from postnatal day 1–2 mice and placed in culture (Fig. 1A). Myelination occurs readily, and is extensive by 10 days *in vitro* (DIV). The gliotoxin lysophosphatidyl choline (LPC) is added at 10 DIV for 15–20 h which leads to almost complete demyelination 24 h after LPC is removed. After 14 additional days in culture (25 DIV), myelin sheaths reappear in conjunction with contactin-associated protein (Caspr) localised at the paranodes around a node, suggestive of a mature myelin internode (Fig. 2E).

The angle of cutting the sections is critical, and requires sagittal sections to minimise nerve fibre damage. Cerebellar slices are much more successful if not detached from the brainstem (Fig. 1B), possibly related to both the maintained connectivity of Purkinje cells and the higher concentration of OPCs in brainstem than cerebellum. Only slices with a maintained neural architecture should be used, for reasons of consistency, which in our hands amounts to about four cerebellar slices attached to brainstem, and three spinal cord slices per mouse. Using these criteria for selection of the slices, the results are consistent between multiple experiments. The concentration and time of LPC addition is also critical as too high a concentration or too long an application also causes axon degeneration. Spinal cord, with its densely packed myelin sheaths (Fig. 1C), requires a longer application of LPC before complete demyelination, probably due to slower LPC penetration (see Materials and methods).

### Characterisation of the cells in the model

In MS, cells at the heart of the demyelinating lesion die, activated microglia/macrophages migrate to the lesion to clear this debris, OPCs migrate to the lesions, proliferate and then differentiate to successfully remyelinate the lesion. OPCs are present in slice cultures as shown by antibody staining to NG2 and are retained after LPC treatment (Fig. 2A). At 25 DIV, we saw mature oligodendrocytes myelinating axons, (MBP positive, Fig. 2B) with clear internodes, heminodes and nodes of Ranvier, flanked by paranodes, identified by tightly localised Caspr expression (Fig. 2E). This is suggestive immunohistochemical evidence that maturation of the myelin sheath occurs, with the formation of specialised nodal and paranodal structures. Cells with a Schwann cell phenotype, the effectors of myelination in the peripheral nervous system, do not myelinate axons in this system as seen by the absence of staining with a periaxin antibody (data not shown).

Activated microglia/macrophages are seen in these cultures, as shown by antibodies to CD68 (Fig. 2C). In MS lesions, macrophages are also recruited from the blood, which is absent in our model, but there are sufficient resident microglia/macrophages to clear most debris. Axonal bulbs, indicative of axonal damage, are sometimes seen after demyelination in our slice model. These are reminiscent of those seen in MS lesions (Dutta and Trapp, 2007) (Fig. 2D).

### OPCs proliferate in response to demyelination

**Fig. 3:**
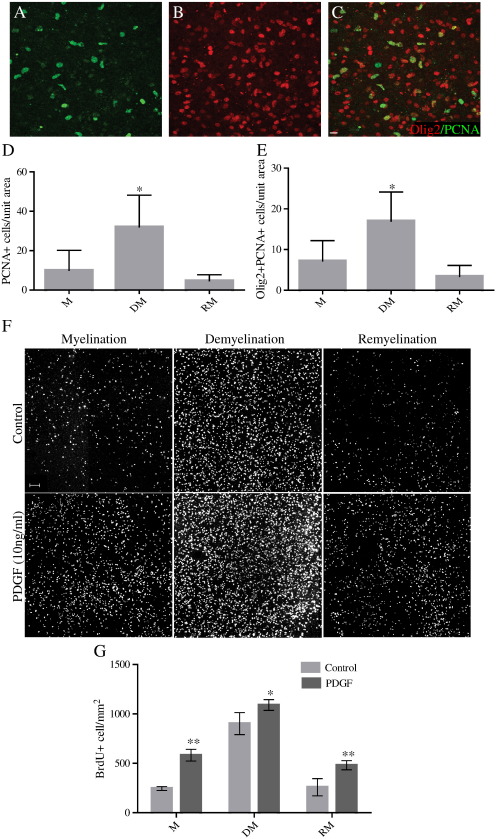
Oligodendroglial cells proliferate in response to demyelination and PDGF. Immunofluorescence for PCNA (green (A)) shows actively proliferating cells in a demyelinated slice, a subset of which are Olig2-positive oligodendroglial cells (red (B)), with merge in (C). Scale bar — 10 μm. D) The number of cells proliferating in cerebellum/brainstem slice cultures, as defined by immunoreactivity against PCNA, increased from 10 DIV (myelination — M), to 12 DIV (demyelination — DM — one day after LPC) and then subsided by 25 DIV (remyelination — RM). E) A subset of these proliferating cells is oligodendroglial cells. Cells double positive for Olig2 and PCNA similarly increased in number from 10 DIV (myelination — M), to 12 DIV (demyelination — DM — one day after LPC) and then subsided by 25 DIV (remyelination — RM). (Mean + S.D., *p < 0.001, ANOVA and Dunnett's Multiple Comparison Test. Unit area = 100,000 μm^2^.) F) Addition of PDGF (10 ng/ml) to the culture medium increased the proliferation of OPCs as assessed by BRDU incorporation (white). (Scale bar 100 μm). G) There is an increase in proliferation of OPCs in slices after addition of PDGF, at each stage, compared to control, as measured by BRDU incorporation at 10 DIV (myelination — M), 12 DIV (demyelination — DM — one day after LPC) and 25 DIV (remyelination — RM) (Mean + S.D., comparisons with control at each time-point. **p < 0.01, *p < 0.05, ANOVA and Dunnett's Multiple Comparison Test. N = 2 experiments. Unit area = 1 mm^2^).

Using antibodies against the marker of proliferation PCNA (proliferating cell nuclear antigen), we showed that treatment with LPC causing demyelination provoked a proliferative response, which subsided with remyelination. The number of proliferating cells in cerebellar/brainstem slices increased 3.2 fold the day after demyelination (DM) (12 DIV) compared to at 10 DIV in control myelinated (M) slices. At 25 DIV (remyelination), the number of proliferating cells had returned to levels indistinguishable from control myelinated slices (M) (Figs. 3A–D). Some of these proliferating cells are oligodendroglial cells as shown by using antibodies to Olig2 and Nkx2.2. The number of cells double positive for Olig2 and PCNA increased 2.4-fold the day after demyelination (DM) (12 DIV) compared to at 10 DIV in control myelinated (M) slices. At 25 DIV (remyelination), the number of proliferating cells had returned to levels indistinguishable from control myelinated slices (M) (Figs. 3A–C, E). Similar results were seen with immunohistochemistry to Nkx2.2 (data not shown). Therefore, demyelination stimulates oligodendroglial proliferation in slice cultures as well as in MS brain. This increase in proliferation of OPCs was also seen by using BrdU incorporation, with a 3.7-fold increase in BrdU-positive OPCs the day after demyelination (DM) (12 DIV), compared to at 10 DIV in control myelinated (M) slices, returning to basal rates at 25 DIV (remyelination) (Figs. 3F and G). Addition of PDGF, a known stimulus of OPC proliferation *in vitro* (Wang et al., 2007) and *in vivo* (Woodruff et al., 2004), further promotes proliferation in slice cultures, both at 10 DIV, with a 2.4-fold increase in the basal rate of proliferation, and after demyelination (12 DIV), with a 1.2-fold increase in numbers of proliferating cells compared to slices in the absence of PDGF (Figs. 3F and G). All cultures are grown in 25% horse serum, containing some PDGF and other growth factors, but even so, addition of exogenous PDGF caused an increase in proliferation over and above that of control cultures.

### Remyelinated fibres have thinner myelin sheaths

**Fig. 4:**
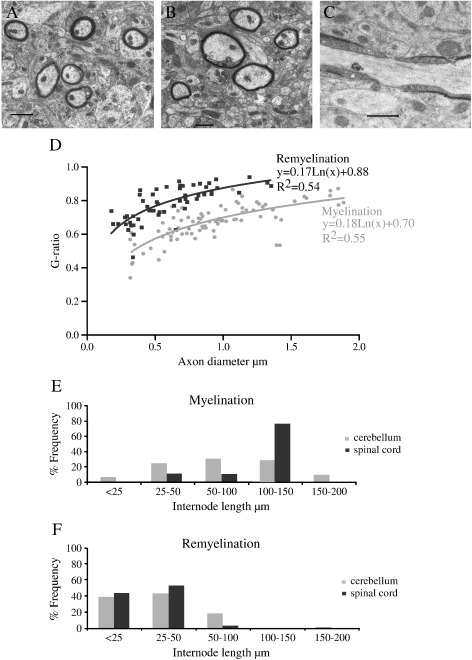
Return of myelin sheaths represents true remyelination. A) Electron micrograph of myelinated axons from cerebellum at 10 DIV. Scale bar — 1 μm. B) Electron micrograph of remyelinated axons from cerebellum at 25 DIV, after LPC treatment on day 10. Note the thinner myelin per axon, especially in relation to their axon diameter. Scale bar — 1 μm. C) Electron micrograph showing a node of Ranvier present in cerebellar cultures that were allowed to remyelinate. Thus, compact myelin forms with mature paranodal structures. Scale bar — 1 μm. D) Quantification of G ratios showing larger G ratios (thinner myelin) for remyelinated axons (dark grey) than that of myelinated axons (pale grey), per axon diameter. These regression lines are significantly different using the maximum likelihood ratio test, p < 0.01 (counted from 6 cerebellar slices per group). E–F) Frequency distribution graphs of internode length in myelinated (E) and remyelinated (F) axons in the cerebellum (pale grey) and spinal cord (dark grey). There is a left shift for remyelinated axons showing that these have shorter internodes. (N = 4 experiments, a minimum of 80 internodes counted per group.) Comparisons of myelination and remyelination frequency-distribution data for both cerebellum and spinal cord is significant with p < 0.01 (Kolmogorov–Smirnov Test).

We considered the possibility that the apparent return of myelin sheaths in our slice system represents myelination of previously unmyelinated nerve fibres. Remyelinated myelin sheaths are thinner and have shorter internodal lengths compared to myelin sheaths formed in development (Perier and Gregoire, 1965; Prineas and Connell, 1979). To investigate whether the return of myelin sheaths after LPC treatment represents genuinely remyelinated fibres, we performed electron microscopy on cerebellar slices, to measure G-ratios (Figs. 4A–C). Myelinated fibres were measured after 10 DIV and “remyelinated” fibres at 25 DIV, with demyelination on day 10. The G ratio represents the ratio of the diameter of the axon to the diameter of the myelinated fibre and allows comparison of myelin thickness for different axon sizes. There was a marked difference between the groups, with “remyelinated” fibres having higher G ratios and thus thinner myelin sheaths. The curves of best fit through the points are shown in Fig. 4D, and these are statistically significantly different using the maximum likelihood ratio test (p < 0.05).

### Remyelinated fibres have shorter internodes

We measured the lengths of the myelin sheaths in both cerebellum and spinal cord, by measuring the distance between paranodes, labelled with anti-Caspr antibodies. We saw that the frequency of short myelin sheaths at 25 DIV (with demyelination on day 10) was much higher than in initial myelination (10 DIV) (Figs. 4E and F). The Caspr staining of paranodes, which reappears in remyelination, plus the compaction of the myelin sheaths seen by electron microscopy, suggests that these remyelinated fibres are mature. Fully formed nodes of Ranvier were also identified (Fig. 4C). Thus, myelin sheaths that reappear after LPC treatment are thinner and shorter than before, pathognomonic of remyelination.

### Automatic quantification of (re)myelination after immunohistochemistry

There is no previously published automated quantification of myelin sheaths. Previously, the amount of myelination has been measured by measuring CNPase activity (Birgbauer et al., 2004; Roth et al., 1995), but this is a marker of differentiation of oligodendrocytes, rather than the presence of myelin sheaths. Similarly, an approach using a beta-galactosidase luminometric assay in a transgenic mouse expressing LacZ under the MBP promoter only measures MBP expression rather than the formation of myelin sheaths (Stankoff et al., 1996). The amount of immunofluorescence staining for MBP has also been used (Ghoumari et al., 2003; Mi et al., 2009; Miron et al., 2010) but this does not allow for the number of axons present (a prerequisite for remyelination), and MBP is present in oligodendrocyte processes and cell bodies as well as myelin sheaths. Recently, it was also described that retinoic acid increased MBP mRNA and protein expression in Schwann cell bodies in the peripheral nervous system, but reduced myelinated internodes (Latasa et al., 2010), indicating that MBP protein alone is not an ideal measure of myelination. A fast, accurate, objective and automatic quantification of the amount of myelin is essential in order to use our model to study factors influencing myelination or remyelination in a high-throughput manner.

**Fig. 5:**
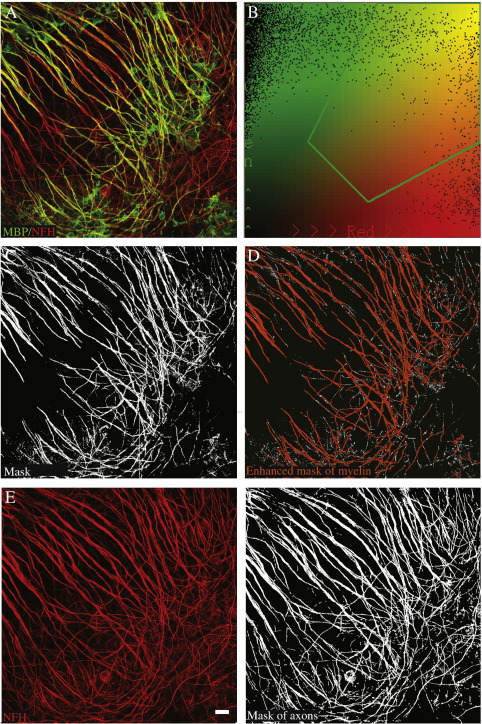
Automatic quantification of (re)myelination. A) Merge of immunofluorescence for MBP (green) and NFH (red) showing overlap. B) Selection of the intensity range corresponding to overlap to be scored as positive (within green line).C) A mask is formed of the overlapping areas (white). D) Overlap areas are coloured orange — excluding predefined very small areas — and quantified, corresponding to the amount of myelin. E) Immunofluorescence for NFH (red) only for the same photograph. F) A mask of all positive pixels for the colour red, corresponding to amount of axons. The Myelination Index (amount of myelin per axon) is determined by dividing the amount of myelin (D) by the amount of axons (F). This quantification takes less than 10 s and avoids counting MBP staining in oligodendrocyte cell bodies (present in green in (A) but excluded in (C)). Scale bar 10 μm.

To determine the amount of myelin per axon, we used immunohistochemistry to stain the structural myelin protein, MBP, in green and the axonal protein NFH in red (Fig. 5A). As MBP also stains oligodendrocyte cell bodies and non-myelinating processes, we measured the overlap between red and green staining at ×40 objective, to quantify only MBP staining overlying axons — the myelin sheaths. We have capitalised on the fact that at this resolution, MBP staining appears to overlie NFH staining, which is lost at higher resolution. We used Image Pro Plus software to write a macro to count pixels which are both red and green above a defined intensity overlap (Fig. 5B) for each layer within a confocal stack, producing a “mask” (Fig. 5C). We exclude extremely small areas of overlap to exclude artefact, forming an “enhanced mask” representing myelinated internodes (Fig. 5D). The number of pixels in the “enhanced mask” is divided by the number of red pixels in the photograph of neurofilament staining only (Figs. 5E and F), obtaining the “Myelination Index”. With this macro, analysis of a stack of images takes around 10 s. This method is fast, objective (not influenced by user preconceptions) and avoids counting most cell bodies.

**Fig. 6:**
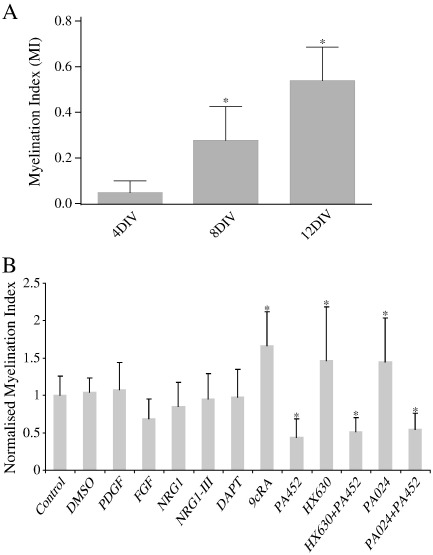
Validation of Myelination Index and model for cerebellar slices. A) The Myelination Index increases linearly between 4 days *in vitro* and 12 days *in vitro*, showing that this method is sensitive enough to detect changes in amount of myelin. *p < 0.01, ANOVA and Dunnett's Multiple Comparison Test. (N = 2 experiments, a minimum of 7 slices counted per condition.) B) Factors previously used to try and alter remyelination were added to the culture medium 24 h after demyelination, and remyelination assessed 14 days after. The data is normalised to the control (no additive) data. Fibroblast growth factor (FGF), Platelet Derived Growth Factor (PDGF), Neuregulin 1 (NRG1), NRG1-III and DAPT (gamma secretase inhibitor) addition showed no difference compared to the controls (control — no additive, PBS added, DMSO added). However, 9 cis-retinoic acid (9cRA) significantly enhances remyelination, and its antagonist PA452 significantly inhibits remyelination. Specific agonists of the RXR receptor, HX630 and PA024 also enhance myelination and this effect is reversed by the use of the antagonist PA452 (Mean + S.D., * p < 0.05 in comparison to control, ANOVA and Dunnett's Multiple Comparison Test) (N = 3 experiments, a minimum of 7 slices counted per condition). This is in concordance with *in vivo* remyelination experiments after demyelination with a myelin toxin in rodents (see Table 1).

To test whether this quantification system is sensitive enough to detect changes in myelination, we used it to measure myelination in the cerebellar slice system at 4 DIV, 8 DIV and 12 DIV. The myelination index increases in a linear way with time, with significant differences between each time point (Fig. 6A) (ANOVA plus Dunnett's multiple comparison test *p < 0.01).

### Validation of the slice model for *in vivo* remyelination

Addition of various factors to *in vitro* co-culture models of developmental myelination have been shown to alter myelination. However, several of these factors fail to affect remyelination in *in vivo* models (summarised and referenced in Table 1). We tested these known factors in our cerebellar slice model for their influence on remyelination, in order to test the fidelity of our slice system to the *in vivo* situation. We tested two factors which promote the proliferation of OPCs and decrease myelination *in vitro* but do not affect myelination *in vivo*: Platelet Derived Growth Factor (PDGF) and Fibroblast Growth Factor (FGF). We used NRG1, NRG1 type 3 and DAPT (which inhibits γ-secretase), as these promote myelination in culture, though are not effective in remyelination *in vivo*. We used 9-cis retinoic acid (9cRA) which promotes remyelination *in vivo*, and the Retinoic acid receptor X (RXR) antagonist PA452, which reduces myelination *in vitro* (Huang et al., 2011). We also used two specific RXR agonists (HX630 and PA025) to show that the effect was specific to the RXR receptor. Each of these factors was added 24 h after the removal of LPC from the culture, and replenished every second day. The myelination index for each factor tested in the slice model is shown in Fig. 6B. As summarised in Table 1, we found that only factors influencing remyelination *in vivo* influenced remyelination in our slice model. Of note, we found that although PDGF addition promoted OPC proliferation (Figs. 3F and G), as found *in vitro* (Wang et al., 2007), it did not change the rate of remyelination in our slice model, which is reflected in its failure to improve remyelination *in vivo* (Woodruff et al., 2004). This suggests that use of our slice model of remyelination, using the myelination index for quantification, is predictive of the *in vivo* effect, both in positively predicting those that have an *in vivo* effect and negatively predicting those that do not.

### Exogenous OPCs added to slice cultures can integrate and form myelin sheaths

Therapies to enhance remyelination aim to either stimulate remyelination by endogenous oligodendroglial cells, or aim to add exogenous cells to fulfil this role. Purified rat OPCs added to mouse cerebellar slices treated with cytosine arabinoside to prevent endogenous myelination, previously achieved some successful myelination presumed to be myelination of mouse axons by rat oligodendroglial cells (Nishimura et al., 1985). We tested whether manipulated exogenous OPCs added to slices can incorporate into the structure, mature and myelinate. We transduced mouse OPCs with lentivirus expressing GFP and pipetted these directly onto cerebellar slices. After 14 days, some GFP-positive OPCs were attached only to the culture membrane, or the edge of the slice, but others penetrated into the slice tissue and myelinated axons (Fig. 2F). We have similarly achieved myelination by adding GFP-positive neurospheres to slices. Thus endogenous and exogenous OPCs form myelin sheaths in our slice model, allowing testing of therapies enhancing both strategies for improving remyelination.

## Discussion

The clinical importance of a validated, robust, high content and moderately-high-throughput system to test putative pro-remyelinating compounds is potentially large. We demonstrate that we have developed such a system enabling us to test the effect of therapies on endogenous remyelination and by the addition of exogenous cells to enhance remyelination.

As in demyelinating lesions *in vivo*, we show that OPCs in our slice system proliferate in response to demyelination, that macrophages are present to clear myelin debris, and that the return of myelin sheaths after demyelination represents compact myelin with characteristics of remyelination. We have automated quantification of myelination, making it objective and reducing analysis time significantly. Although truly high throughput screening would be impossible in this system, it allows us to test tens of compounds over a period of a month, in comparison to several years for testing the same number of compounds *in vivo*. Only the most promising strategies could then be tested *in vivo*, saving much in terms of time and resources.

On testing the system with factors known to alter myelination *in vitro*, some of which also alter remyelination *in vivo*, the slice model results were consistent with the *in vivo* remyelination model. This is in spite of using early postnatal mouse brain grown for 10 days in culture, which may be more plastic than adult brain. Remyelination shares similarities with myelination, but also has significant differences as remyelination occurs on axons which are demyelinated (which may change them) in the presence of inflammatory debris and tissue injury.

Cerebellar slice cultures have the advantage that most myelinated axons are of one type — Purkinje cell axons. However, brain stem and spinal cord slices myelinate more robustly than cerebellum in culture and contain a mixture of fibre types and diameters. This is perhaps more reflective of the situation in both development and MS, which is not nerve fibre type specific. Spinal cord contains a pool of OPCs from different developmental sources compared to brain (Vallstedt et al., 2005) and is linear, and may be used for OPC migration experiments using local sources of chemotactic molecules. Sagittal slices of cerebral cortex have also been cultured, and the corpus callosum also shows myelination (Supplementary Fig. 1).

As our slice system uses mouse tissue, it can be used to examine remyelination in slices from transgenic animals. This allows quicker assessment of their remyelination capacity, but also more detailed assessment of the biology as it can be easily assessed at different time-points and even used in combination with time-lapse microscopy to watch events in real-time. We have shown that lentiviruses can be used to manipulate the biology of the slices. Lentiviral transduction of slices is highly efficient and can be used to add genes under cell-specific promoters, or reduce protein expression using mi/si/shRNA technology. In the case of conditional and inducible transgenic mice, viruses could be used to induce Cre expression and excise the gene of interest, or the system could be induced with tamoxifen/doxycycline to investigate the biology *ex vivo*, before deciding to invest much effort, time and money in breeding mice for lengthy *in vivo* experiments. Semliki Forest viruses have also successfully been used to transduce oligodendrocytes in mouse hippocampal slice cultures, enabling them to be tracked by live imaging (Haber et al., 2009).

We have also shown that OPCs can be manipulated in culture with lentiviruses, added to slices, and migrate into slices to myelinate axons. This is a proof of principle that the action of exogenous cells can be studied in our system. We predict that OPCs derived from embryonic/ induced pluripotent stem cells will incorporate similarly and so this system can also be used to screen stem cell based therapies to enhance remyelination.

The pathology of MS is complex and exclusively human, so no model of the disease is perfect. Our system models remyelination, a key therapeutic target in MS for prevention of axonal degeneration. We aimed to model active demyelinating MS plaques, which often have some propensity to remyelinate, rather than chronic MS plaques which do not. Our system does not model the inflammatory pathology of MS, as although microglia are present, there is no blood supply, and so T and B cell infiltrates are not present. Thus, its use is similar to that of the *in vivo* MS models using myelin toxins to cause demyelination and study remyelination, rather than EAE. The advantage of these systems is that remyelination can be studied without the complexity of ongoing inflammation.

We present this automated slice system as an appropriate model of *in vivo* remyelination in the rodent CNS, and as a model of pathology in MS. We suggest that it can be used as an intermediate step between *in vitro* experiments and undertaking lengthy *in vivo* studies, to select the most promising compounds, though clearly not replacing the latter. Using the slice system in this way will reduce live animal usage and has a large potential for studying therapeutics and biology of remyelination and even neuroprotection, accelerating the translation of compounds from mouse to man.

The following is the supplementary material related to this article.

Supplementary Fig. 1 Myelination in corpus callosum in cerebral hemisphere slices. An oligodendrocyte in the corpus callosum of a cerebral hemisphere slice myelinating axons at 10 DIV. (NFH, red, MBP, green). Scale bar 10 μm.

## Figures and Tables

**Table 1:** Comparison of effect of factors on myelination and remyelination.

Factor	Action on OPCs	Myelination *in vitro* co-cultures	Remyelination in slices	Remyelination *in vivo* (myelin toxin model)
PDGF	↑ Proliferation	↓ Myelination (Wang et al., 2007)	↔	↑ OPC numbers but remyelination ↔ (Woodruff et al., 2004)
FGF2	↑ Proliferation	↓ Myelination (Wang et al., 2007)	↔	Not known
NRG1	↑ Proliferation and survival	↑ Myelination (Wang et al., 2007)	↔	Remyelination ↔ (Penderis et al., 2003)
Inhibition of γ-secretase/Notch-1 ablation	↑ Differentiation	↑ Myelination (Watkins et al., 2008)	↔	Remyelination ↔ with Notch-1 ablation (Stidworthy et al., 2004)
9-cis retinoic acid	↑ Differentiation	↔ Myelination (Huang et al., 2011)	↑	↑ Remyelination (Huang et al., 2011)
