# Supplementary figures and images for: Central nervous system remyelination in culture — A tool for multiple sclerosis research
Source: Exp Neurol. 2011 Jul;230(1-2):138–48. doi: 10.1016/j.expneurol.2011.04.009 (PMC3117145; doi:10.1016/j.expneurol.2011.04.009)

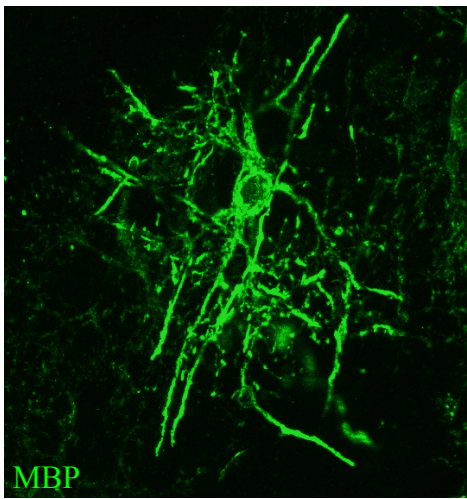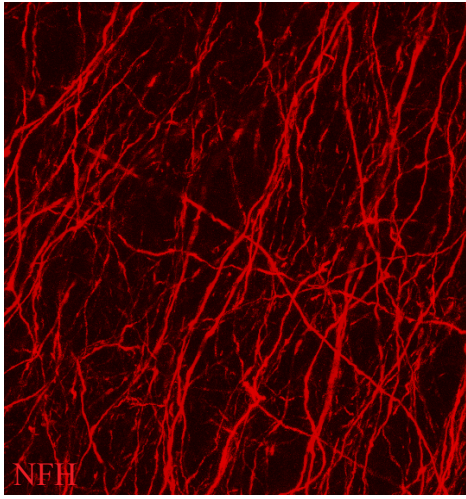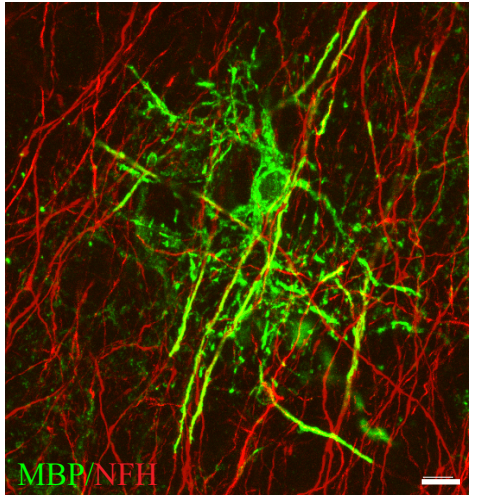

Supplement: Supplementary Fig. 1 — Myelination in corpus callosum in cerebral hemisphere slices. An oligodendrocyte in the corpus callosum of a cerebral hemisphere slice myelinating axons at 10 DIV. (NFH, red, MBP, green). Scale bar 10 μm. [file mmc1.pdf]
